# Epidemiology of carbapenem-resistant organisms in Alameda County, California, 2019–2021

**DOI:** 10.1017/ash.2024.33

**Published:** 2024-04-29

**Authors:** Rachel Marusinec, Munira Shemsu, Tyler Lloyd, Brendan M. Kober, Dustin T. Heaton, Jade A. Herrera, Misha Gregory, Vici Varghese, Joelle Nadle, Kavita K. Trivedi

**Affiliations:** 1 Alameda County Health, Public Health Department, Division of Communicable Disease Control and Prevention, San Leandro, CA, USA; 2 Alameda County Health, Public Health Department, Public Health Laboratory, Oakland, CA, USA; 3 California Emerging Infections Program, Oakland, CA, USA

## Abstract

**Objective:**

Carbapenem-resistant organisms (CROs) are an urgent health threat. Since 2017, Alameda County Health Public Health Department (ACPHD) mandates reporting of carbapenem-resistant Enterobacterales (CRE) and encourages voluntary reporting of non-CRE CROs including carbapenem-resistant *Acinetobacter baumannii* (CRAB) and carbapenem-resistant *Pseudomonas aeruginosa* (CRPA). Surveillance data from ACPHD were analyzed to describe the epidemiology of CROs and target public health interventions.

**Methods:**

Healthcare facilities in Alameda County reported CRO cases and submitted isolates to ACPHD to characterize carbapenemase genes; deaths were identified via the California Electronic Death Registration System. CRO cases with isolates resistant to one or more carbapenems were analyzed from surveillance data from July 2019 to June 2021.

**Results:**

Four hundred and forty-two cases of CROs were reported to Alameda County from 408 patients. The county case rate for CROs was 29 cases per 100,000 population, and cases significantly increased over the 2-year period. CRPA was most commonly reported (157 cases, 36%), and cases of CRAB increased 1.83-fold. One-hundred eighty-six (42%) cases were identified among residents of long-term care facilities; 152 (37%) patients had died by January 2022. One hundred and seven (24%) cases produced carbapenemases.

**Conclusions:**

The high burden of CROs in Alameda County highlights the need for continued partnership on reporting, testing, and infection prevention to limit the spread of resistant organisms. A large proportion of cases were identified in vulnerable long-term care residents, and CRAB was an emerging CRO among this population. Screening for CROs and surveillance at the local level are important to understand epidemiology and implement public health interventions.

## Background

Carbapenem-resistant organisms (CROs) are considered urgent national health threats^1^ and are part of the global health threat of antimicrobial resistance with an estimated 243,000 deaths worldwide attributable to carbapenem-resistant pathogens in 2019.^2^ In the United States, carbapenem-resistant Enterobacterales (CRE) and carbapenem-resistant *Acinetobacter* spp. were estimated to account for 13,100 and 8,500 hospitalizations in 2017, respectively.^1^

One of the mechanisms of carbapenem resistance is the carriage of carbapenemase genes. In the United States, five of the most common genes conferring resistance are *Klebsiella pneumoniae* carbapenemase (KPC), New Delhi metallo-beta-lactamase (NDM), oxacillinase-48 (OXA-48), Verona integron-encoded metallo-beta-lactamase (VIM), and imipenemase (IMP).^3,4^ In addition to clonal expansion, the spread of carbapenemase genes can occur by plasmid-mediated horizontal gene transfer,^5,6^ affecting transmissibility. Therefore, whether an outbreak or infection is due to a carbapenemase-producing CRO (CP-CRO) or a non-carbapenemase-producing CRO (non-CP-CRO) has implications for prevention and control.

In June 2017, due to the increasing incidence of CRE in the San Francisco Bay Area, Alameda County, with a population of over 1.6 million residents,^7^ issued a health officer order requiring the reporting of CRE—specifically *Escherichia coli*, *Klebsiella* spp., and *Enterobacter* spp.—and submission of isolates to the Alameda County Health Public Health Department (ACPHD).^8^ Prior to the coronavirus disease 2019 (COVID-19) pandemic, health officer orders were used sparingly; the ACPHD order required more than what was or still is shared with other local public health and state public health surveillance; of note, the California Emerging Infections Program (CEIP) has been conducting active laboratory-based surveillance for CREs only in our county and others since 2017.^9^ From the initial order to July 2019, healthcare facilities submitted 168 CRE isolates, of which 26% were CP-CROs (unpublished data). In this analysis, we summarize reported CROs from July 2019 to July 2021, just prior to and during the COVID-19 pandemic, to understand the trends in the incidence of CROs in Alameda County.

## Methods

### Definitions

A patient was defined as a person who tested positive for a CRO from July 2019 to June 2021. A case was defined as the isolation of a specific CRO from a patient. An isolate was a bacterial sample from a body source that tested positive for a CRO. For analysis purposes, a surveillance sample was defined as a CRO isolate from a rectal sample,^10^ and a clinical sample was a CRO isolate from a non-rectal sample.

An isolate was considered carbapenem-resistant if it was any of the following: (1) resistant to any carbapenem, with a minimum inhibitory concentration (MIC) of ≥4μ g/mL for doripenem, imipenem, or meropenem or ≥2 μg/mL for ertapenem; (2) documented to produce a carbapenemase, demonstrated using a Centers for Disease Control and Prevention (CDC)-accepted test (modified Hodge, Carba NP, modified carbapenem inactivation method (mCIM), and mCIM with EDTA-CIM (eCIM)); or (3) demonstrated to possess a carbapenemase gene using a CDC-accepted test (polymerase chain reaction (PCR), whole genome sequencing (WGS)).^8^ An organism was considered a CP-CRO if it was identified to have carbapenemase activity or carbapenemase gene(s) by either mCIM, PCR, WGS, or Carba NP. An organism was considered a non-CP-CRO if it was identified to not have carbapenemase activity or carbapenemase genes by either mCIM, WGS, or Carba NP.

Facility type was defined as the type of facility the patient was admitted to when they tested positive for a CRO unless their residence was noted to be a long-term care facility (LTCF). Facility types categorized as LTCFs include skilled nursing facilities (SNFs), long-term acute care hospitals (LTACHs), and ventilator-equipped skilled nursing facilities (vSNFs).

### Data ascertainment

Pursuant to the Alameda County Health Officer mandate, medical providers are required to notify ACPHD of specific CRE cases and submit their bacterial isolates to the Alameda County Health Public Health Laboratory (ACPHL)^8^; voluntary reporting and isolate submission of non-CRE CROs are also encouraged. CRE and CRO cases are reported through electronic laboratory reporting via the California Reportable Disease Information Exchange (CalREDIE), through submission of confidential morbidity reports, or by directly contacting ACPHD. Upon receiving initial case reports, further investigation data is entered into CalREDIE by public health disease investigators for reporting to the California Department of Public Health. Deaths were identified by matching case name, date of birth, and zip code to the California Comprehensive Death File, from the California Electronic Death Registration System. Cases reported through CalREDIE were conducted through passive surveillance, while active surveillance was also performed through the Antibiotic Resistance Lab Network (ARLN) for select facilities experiencing a CRO outbreak to detect the presence of carbapenemase genes and/or carbapenemase activity.

Isolates submitted to ACPHL were tested for carbapenemase genes by WGS using the Illumina MiSeq platform (Illumina, San Diego, CA, USA).^11^ Other tests performed by identifying facility clinical labs include Carba-R (Cepheid, Sunnyvale, CA, USA), WGS (Illumina, San Diego, CA, USA), OXA-allele PCR, mCIM, and Carba NP (bioMérieux, Marcy-l'Étoile, France). OXA-allele detection for all OXA genes including carbapenemases such as OXA-235-like was performed using a Lab Developed Test PCR assay by the ARLN and by ACPHL using WGS.

### Analysis

CRO cases identified from July 2019 to June 2021 were analyzed using R Statistical Software (v4.0.2; R Core Team, 2020). Odds ratios (ORs) were calculated with the R package epitools (v0.5-10.1; Aragon, 2020) using the Wald method with an alpha of 0.05. Poisson regression was used to determine the trend in CRO incidence over time. Graphs were created using the R package ggplot2 (H. Wickham, 2016). Bed counts were obtained from the Licensed Healthcare Facility Listing from California Health and Human Services’ Department of Healthcare Access and Information.^12^ Facility case rates were calculated using the number of cases divided by the number of beds in facilities. Post-analysis completeness of reporting was verified by a sensitivity analysis using active population-based laboratory surveillance data collected by CEIP^13^ for CRE only and case numbers requested from a local short-term acute care hospital (STACH) and LTCF to determine if we captured all CRO cases identified during this period.

Collection and reporting of CROs in Alameda County is a public health surveillance activity authorized by the public health authority and therefore not considered human subjects research.

## Results

During the 2-year study period, 442 cases met the CRO case definition from 408 patients; 651 bacterial isolates were tested from these patients. Thirty-one patients had more than one CRO. Patient characteristics are described in Table 1. Two-hundred forty-five (60%) patients were over the age of 60. Half of the patients (206, 50%) were from STACHs, and 170 patients (42%) were from an LTCF. By six months after the study period, 152 (37%) of the patients had died.

**Table 1.** Characteristics of patients with an identified carbapenem-resistant organism

Patient characteristic	N = 408^a^
Age group (y/o)	
<18	5 (1.2%)
18–30	22 (5.4%)
31–40	36 (8.8%)
41–50	31 (7.6%)
51–60	69 (17%)
61–70	98 (24%)
71–80	91 (22%)
81+	56 (14%)
Race/ethnicity	
African American or Black	51 (13%)
Asian	37 (9.1%)
Hispanic or Latino/a/x	54 (13%)
White	80 (20%)
Other/multiple races	42 (10%)
Unknown	144 (35%)
Gender	
Female	165 (40%)
Male	227 (56%)
Other or unknown	16 (4.0%)
Facility type^b^	
Long-term acute care hospital	91 (22%)
Short-term acute care hospital	206 (50%)
Skilled nursing facility	20 (4.9%)
Ventilator-equipped skilled nursing facility	59 (14%)
Outpatient	20 (4.9%)
Other^c^	12 (2.9%)

a *n* (%).

b Facility where the patient was located when the organism was first identified, or reporting facility.

c Includes in-home care and lab facilities.

The county case rate for CROs over the 2-year period was 29 cases per 100,000 population, and the county case rate for CP-CROs was 7 cases per 100,000 population. The most common species among cases was carbapenem-resistant *Pseudomonas aeruginosa* (CRPA), while the least common was carbapenem-resistant *Acinetobacter baumannii* (CRAB) (Table 2). Cases of reported CRAB increased by 183% in the second year of the study period compared with the first, and CRAB had the highest proportion of carbapenemase-producing cases (59%). Twenty-four percent (107) of all 442 cases were CP-CROs. CRPA and *Enterobacter* spp. had the lowest proportion of carbapenemase-producing cases (Table 2).

**Table 2.** Carbapenem-resistant organism species by carbapenemase-producing status among cases (n = 442), Alameda County, California, July 2019–June 2021

Isolated species	Carbapenemase-producing, n (%)	Non-carbapenemase-producing, n (%)	Not tested, n (%)
Overall	107 (24%)	277 (63%)	58 (13%)
Reportable carbapenem-resistant Enterobacterales^a^, n = 216	61 (28%)	133 (62%)	22 (10%)
Carbapenem-resistant *E. coli*, n = 55	18 (33%)	31 (56%)	6 (11%)
Carbapenem-resistant *Enterobacter* spp., n = 73	7 (10%)	59 (80%)	7 (10%)
Carbapenem-resistant *Klebsiella* spp., n = 88	36 (41%)	43 (49%)	9 (10%)
Carbapenem-resistant *Acinetobacter baumannii*, n = 46	27 (59%)	4 (9%)	15 (33%)
Carbapenem-resistant *Pseudomonas aeruginosa*, n = 157	15 (10%)	129 (82%)	13 (8%)
Other CRO^b^, n = 23	4 (17%)	11 (48%)	8 (35%)

Note. CRO, carbapenem-resistant organism.

a *E. coli*, *Klebsiella* spp., and *Enterobacter* spp.

b Includes *Bordetella trematum* (n = 1), *Citrobacter freundii* (n = 2), *Lelliottia amnigena* (n = 1), *Morganella morganii* (n = 1), *Pantoea agglomerans* (n = 1), *Proteus mirabilis* (n = 6), *Providencia stuartii* (n = 4), *Raoultella ornithinolytica* (n = 1), *Serratia marcescens* (n = 5), and *Stenotrophomonas maltophilia* (n = 1).

**Figure 1.**
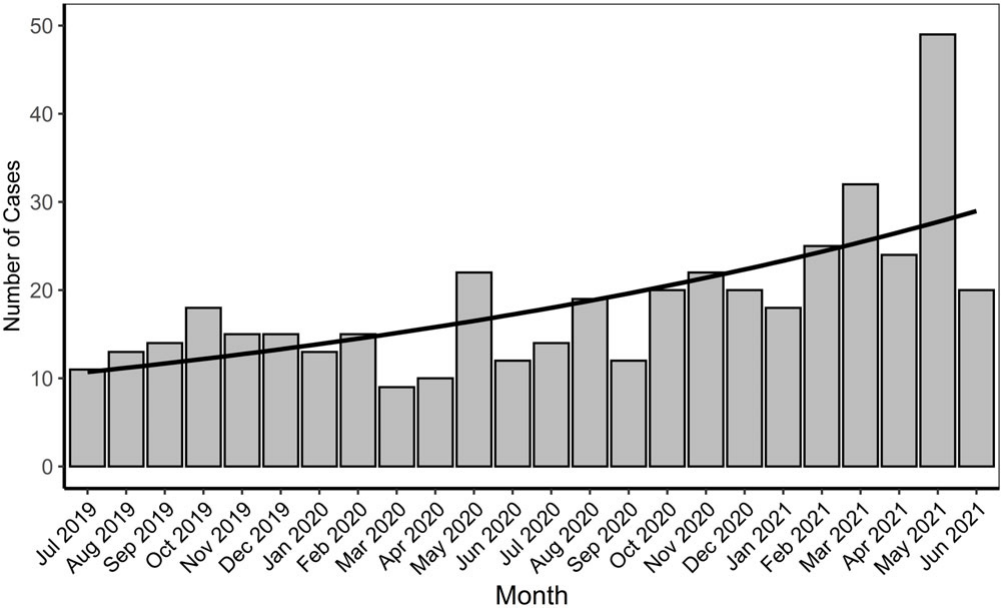
Number of carbapenem-resistant organism cases reported by month, Alameda County, California, July 2019–June 2021. Trend line calculated by Poisson regression, *P* < .05.

Reported CRO cases increased significantly from the beginning of the study period to the end (Figure 1). Reportable CRE cases increased, although not significantly, while both the number of CRPA and CRAB cases significantly increased (*P* < .05). Three outbreaks occurred among three different LTCFs during the study period—beginning in May 2020, February 2021, and late April 2021, corresponding with months of increased cases. Among the outbreak facilities, the 2-year incidence rate ranged from 16 to 107 cases per 100 beds. The 2-year incidence rate for all Alameda County healthcare facilities was 4.9 cases per 100 beds. CP-CRO cases were more likely to be found among LTCFs than acute care facilities (OR, 1.49; 95% CI, 0.93–2.34), and CP-CRO cases were significantly more likely to be found among vSNFs than other facilities (OR, 2.29; 95% CI, 1.3–4.0).

Among cases, the most identified gene was KPC (33, 7.5%, Table 3), primarily identified among reportable CRE species. NDM was the second most identified carbapenemase (28 cases, 6.3%) and most common among CRAB cases, along with OXA-235-like genes (Table 3). Of the six NDM CRAB cases identified in our county, three (50%) were epidemiologically linked to facilities outside the county where a regional NDM CRAB outbreak began, and the two CRAB cases with an OXA-23-like/NDM resistance gene combination were detected at separate facilities in our county, indicating transmission between facilities.

**Table 3.** Identified carbapenemase genes, specimen sources, and species for the 442 CRO cases, Alameda County, California, July 2019–June 2021

Characteristic	Reportable CRE^a^, N = 216^b^	Carbapenem-resistant *Acinetobacter baumannii*, N = 46^b^	Carbapenem-resistant *Pseudomonas aeruginosa*, N = 157^b^	Other CRE/CRO, N = 23^b^	Total, N = 442^b^
Carbapenemase gene					
KPC	31 (14%)	0 (0%)	1 (0.6%)	1 (4.3%)	33 (7.5%)
NDM	19 (8.8%)	6 (13%)	2 (1.3%)	1 (4.3%)	28 (6.3%)
VIM	0 (0%)	0 (0%)	12 (7.6%)	0 (0%)	12 (2.7%)
OXA-48-like	8 (3.7%)	1 (2.2%)	0 (0%)	0 (0%)	9 (2.0%)
IMP	0 (0%)	0 (0%)	2 (1.3%)	1 (4.3%)	3 (0.7%)
OXA-235-like	0 (0%)	9 (20%)	0 (0%)	0 (0%)	9 (2.0%)
OXA-23-like	0 (0%)	7 (15%)	0 (0%)	0 (0%)	7 (1.6%)
Other genes^c^	2 (0.9%)	3 (6.5%)	0 (0%)	1 (4.3%)	6 (1.4%)
No carbapenemase genes identified	119 (55%)	3 (6.5%)	129 (82%)	9 (39%)	260 (59%)
Not tested for specific carbapenemase genes	41 (19%)	20 (44%)	14 (8.9%)	10 (44%)	85 (19%)
Specimen source^d^					
Blood	17 (7.9%)	0 (0%)	0 (0%)	2 (8.7%)	19 (4.3%)
Perirectal/rectal/fecal swab	33 (15%)	6 (13%)	11 (7.0%)	3 (13%)	53 (12%)
Respiratory	30 (14%)	23 (50%)	108 (69%)	7 (30%)	168 (38%)
Urine	109 (50%)	5 (11%)	23 (15%)	6 (26%)	143 (32%)
Wound	10 (4.6%)	8 (17%)	10 (6.4%)	2 (8.7%)	30 (6.8%)
Other^e^	17 (7.9%)	2 (4.3%)	5 (3.2%)	2 (8.7%)	26 (5.9%)
Not specified	0 (0%)	2 (4.3%)	0 (0%)	1 (4.3%)	3 (0.7%)

Note. CRE, carbapenem-resistant Enterobacterales; CRO, carbapenem-resistant organism; KPC, *Klebsiella pneumoniae* carbapenemase; NDM, New Delhi metallo-beta-lactamase; OXA-48, oxacillinase; VIM, Verona integron-encoded metallo-beta-lactamase; IMP, imipenemase.

a *E. coli*, *Enterobacter* spp., *and Klebsiella* spp.

b n (%).

c Includes OXA-181 and OXA-24/40.

d The specimen site of the first isolate collection is reported.

e Includes abscess (n = 7), tissue (n = 4), abdominal fluid (n = 4), bone (n = 4), skin (n = 2), jaw (n = 1), blood and respiratory (n = 1), gallbladder (n = 1), vaginal (n = 1), and vulva (n = 1) sites.

Bacterial sample sites differed by CRO species. Most CRPA (108, 69%) and half of CRAB (23, 50%) cases had initial isolates from respiratory specimens, while 109 (50%) of reportable CRE cases were identified from urine samples. Eight (17%) CRAB cases were identified from wound samples, a higher percentage than both CRPA and CRE (Table 3).

The sensitivity analysis revealed that for mandated-reportable CRE, our analysis included 154 of 166 (93%) cases actively found by CEIP and 11 of 13 (76%) isolates on record for a local STACH. For voluntary CRO reporting, our analysis included 12 of 20 (60%) cases from a local SNF and 6 of 24 (25%) isolates on record for those cases.

## Discussion

The number of reported CRO cases in Alameda County increased from July 2019 to July 2021 (Figure 1), similar to increases observed elsewhere in recent years.^4,14^ Twenty-four percent of the CRO cases over the 2-year period were carbapenemase-producing, and 28% of reportable CRE cases were carbapenemase-producing. This is higher than the percentage found during the same time frame in Tennessee (14%–26%)^14^ and within the range found by the Multi-site Gram-negative Surveillance Initiative from 2011 to 2015 (16%–91%).^15^ In line with US trends for CP-CRE,^15^ KPC was the most common carbapenemase found followed by NDM (Table 3). The percentage of CP-CRE in a population varies greatly by geography,^16^ highlighting the need for understanding and sharing local epidemiology.

Most cases were identified from respiratory or urine samples and less than 15% from rectal swabs, an indirect indication that a high proportion of cases identified were clinical samples rather than surveillance samples. CRE isolates were most likely to be isolated from urine, while CRPA and CRAB isolates were most likely to be isolated from respiratory samples. This may have implications for screening patients when there is an outbreak of a specific organism. Although rectal swabs are often used for screening, other sites might be considered for better detection, depending on the organism being screened. Although studies have found the groin and rectal/perirectal regions to be most sensitive for CRE and MDR Enterobacterales,^17,18^ sensitivity of different screening sites is higher for other CROs, such as the skin or buccal mucosa and the skin for CRAB,^19^ while for others, like CRPA, screening site specificity is not well documented. Further studies would be warranted to determine if urine and rectal culture results tend to be concordant to suggest urine as a screening site for CRE, as well as respiratory and rectal cultures as screening sites for CRPA and CRAB.

There are currently many more studies assessing the epidemiology of CRE than CRAB in the United States, and the emergence of CRAB and 1.83-fold increase in our county without a reporting mandate highlights the importance of conducting surveillance for this organism. Although the CDC shows a decrease in hospitalized carbapenem-resistant *Acinetobacter* cases from 2012 to 2017,^1^ there is still a concern around CRAB due to the high percent producing carbapenemases.^20^ Our analysis found that 59% of CRAB cases were carbapenemase-producing (Table 2) and that the most common carbapenemase genes conferring resistance among CRAB cases were NDM (6/46, 13%), OXA-23-like (7/46, 15%), and OXA-235-like (9/46, 20%) (Table 3). This differs from the gene breakdown of CRAB isolates submitted to the CDC from 2013 to 2017: 49% of which had OXA-23-like, 5% having OXA-235-like, and none having NDM;^20^ likely due to three LTCFs experiencing outbreaks during the study period. Other CROs were also epidemiologically linked to cases in other facilities, demonstrating likely transmission between facilities.

With regard to attribution, half of the patients were at an STACH, while 42% were at an LTCF when their first CRO organism was identified. Although other studies of CRE in the United States have found a majority of cases identified from STACHs,^4,21^ incidence rates have been found to be higher among LTACHs than STACHs^22^ and higher prevalence rates among LTCFs compared with STACHs.^16^ Transmission of CRE in LTCFs has been well documented,^23,24^ and prior stays in high-acuity LTCFs have been identified as risk factors for CRE carriage at hospital admissions,^25^ indicating LTCFs as a reservoir for CRO colonization.^16^

Of particular concern are outbreaks and transmission of CP-CROs at LTCFs. The spread of carbapenemase genes via plasmids has been implicated in healthcare facility outbreaks.^26^ Our analysis found that cases identified at LTCFs were more likely to be CP-CRO cases than those at acute care facilities, although the increased odds were not significant. The vSNFs were found to have significantly higher odds of CP-CRO cases than other facility types. This is similar to other analyses that have identified vSNFs and time on ventilators as risk factors for CP-CROs during outbreaks.^23,25^ Due to the heightened potential for transmission and effect on infection control, it is important to test for carbapenemase genes in the vSNF setting. Focusing limited public health resources in high-risk settings such as vSNFs can be extremely impactful in limiting the spread of antibiotic resistance.

Due to the scale of the three vSNF and LTACH outbreaks during the study period, Alameda County worked with these facilities to implement a multi-interventional approach similar to other successful outbreak investigations.^27^ Facilities closed to new admissions during the outbreak, implemented contact precautions for CRO patients, and ACPHD recommended strategic cohort isolation of patients and staff, provided education to staff, conducted onsite assessment and adherence monitoring to infection control and prevention practices, and conducted point prevalence surveys every two weeks until the outbreaks resolved. Local public health must coordinate regional surveillance and response to prevent and control vSNF/LTACH outbreaks.

This analysis is subject to several limitations. First, most cases occurred during the COVID-19 pandemic during which there was limited staff capacity at the local public health department and at healthcare facilities that may have affected the completeness of reporting. In addition, this analysis includes data for both mandatory and voluntarily reportable diseases. The sensitivity analysis demonstrated relatively high completeness for CRE, but isolate submission for CRE cases was not as complete. Voluntary non-CRE CROs had lower completeness results. Only 60% of non-CRE CROs on record at a local SNF were reported to the health department and included in this analysis, and only one-quarter of those cases had isolates submitted, which may have biased the distribution of CROs detected. CROs from facilities experiencing outbreaks may be overrepresented. These data demonstrate that CROs are an increasing issue and worthy of surveillance and should be considered for mandated reporting beyond CRE. Furthermore, these results data support how mandated reporting allowed for more complete case finding than voluntary reporting.

In addition, because the data analyzed are surveillance data, we are limited to what is entered into CalREDIE to determine the facility the patient was at when they tested positive for a CRO. This may lead to some misclassification of facility type associated with a case, as a case may be tested and reported by an STACH for a patient residing in an LTCF, without the STACH indicating that the patient was transferred from an LTCF. To reduce this misclassification, we utilized patient addresses in the surveillance system to determine if they resided at an LTCF.

In conclusion, the number of reported CROs increased significantly from July 2019 to June 2021 in Alameda County. CRAB cases saw a large increase, driven by regional outbreaks. Like other US jurisdictions, most CRO cases were identified in STACHs, in part due to compliance with local reporting requirements and prior stays at LTCFs that may not have been easily ascertained. LTCFs such as vSNFs are facilities at high risk for outbreaks that can amplify the spread of emerging genes, and public health resources should be focused on reducing transmission of CROs in this setting. These data could also help target where screening for specific CROs occurs (respiratory vs urine samples) to improve effectiveness with limited resources.

Finally, Alameda County’s local health officer order allowed the local public health department to act on multiple CRO outbreaks and partner with facilities to implement a multi-interventional approach to limit the transmission of CROs. It is important to focus on these highly resistant organisms because many of the patients with these CROs had poor clinical outcomes, including death, highlighting the significant health impact of these conditions.
